# *Decoy receptor 1* (*DCR1)* promoter hypermethylation and response to irinotecan in metastatic colorectal cancer

**DOI:** 10.18632/oncotarget.18702

**Published:** 2017-06-27

**Authors:** Linda J.W. Bosch, Geert Trooskens, Petur Snaebjornsson, Veerle M.H. Coupé, Sandra Mongera, Josien C. Haan, Susan D. Richman, Miriam Koopman, Jolien Tol, Tim de Meyer, Joost Louwagie, Luc Dehaspe, Nicole C.T. van Grieken, Bauke Ylstra, Henk M.W. Verheul, Manon van Engeland, Iris D. Nagtegaal, James G. Herman, Philip Quirke, Matthew T. Seymour, Cornelis J.A. Punt, Wim van Criekinge, Beatriz Carvalho, Gerrit A. Meijer

**Affiliations:** ^1^ Department of Pathology, VU University Medical Center, Amsterdam, The Netherlands; ^2^ Department of Pathology, The Netherlands Cancer Institute, Amsterdam, The Netherlands; ^3^ Department of Mathematical Modelling, Statistics and Bioinformatics, Ghent University, Ghent, Belgium; ^4^ Department of Epidemiology and Biostatistics, VU University Medical Center, Amsterdam, The Netherlands; ^5^ Pathology and Tumour Biology, University of Leeds, Leeds, UK; ^6^ Department of Medical Oncology, University Medical Center Utrecht, Utrecht, The Netherlands; ^7^ Department of Internal Medicine, Jeroen Bosch Hospital, ‘s-Hertogenbosch, The Netherlands; ^8^ MDxHealth, SA, Liège, Belgium; ^9^ Genomics Core Facility, UZ Leuven, Leuven, Belgium; ^10^ Department of Oncology, VU University Medical Center, Amsterdam, The Netherlands; ^11^ Department of Pathology, GROW - School for Oncology and Developmental Biology and Maastricht University Medical Center, Maastricht, The Netherlands; ^12^ Department of Pathology, Radboud University Nijmegen Medical Center, Nijmegen, The Netherlands; ^13^ Department of Medicine, University of Pittsburgh, Pittsburgh, PA, USA; ^14^ St James's Institute of Oncology, St James's University Hospital, Leeds, UK; ^15^ Department of Medical Oncology, Academic Medical Center, Amsterdam, The Netherlands

**Keywords:** TNFRSF10C, biomarker, predictive, chemotherapy, CAIRO

## Abstract

Diversity in colorectal cancer biology is associated with variable responses to standard chemotherapy. We aimed to identify and validate DNA hypermethylated genes as predictive biomarkers for irinotecan treatment of metastatic CRC patients.

Candidate genes were selected from 389 genes involved in DNA Damage Repair by correlation analyses between gene methylation status and drug response in 32 cell lines. A large series of samples (n=818) from two phase III clinical trials was used to evaluate these candidate genes by correlating methylation status to progression-free survival after treatment with first-line single-agent fluorouracil (Capecitabine or 5-fluorouracil) or combination chemotherapy (Capecitabine or 5-fluorouracil plus irinotecan (CAPIRI/FOLFIRI)).

In the discovery (n=185) and initial validation set (n=166), patients with methylated *Decoy Receptor 1* (*DCR1)* did not benefit from CAPIRI over Capecitabine treatment (discovery set: HR=1.2 (95%CI 0.7-1.9, *p*=0.6), validation set: HR=0.9 (95%CI 0.6-1.4, *p*=0.5)), whereas patients with unmethylated *DCR1* did (discovery set: HR=0.4 (95%CI 0.3-0.6, *p*=0.00001), validation set: HR=0.5 (95%CI 0.3-0.7, *p*=0.0008)). These results could not be replicated in the external data set (n=467), where a similar effect size was found in patients with methylated and unmethylated *DCR1* for FOLFIRI over 5FU treatment (methylated *DCR1*: HR=0.7 (95%CI 0.5-0.9, *p*=0.01), unmethylated *DCR1*: HR=0.8 (95%CI 0.6-1.2, *p*=0.4)).

In conclusion, *DCR1* promoter hypermethylation status is a potential predictive biomarker for response to treatment with irinotecan, when combined with capecitabine. This finding could not be replicated in an external validation set, in which irinotecan was combined with 5FU. These results underline the challenge and importance of extensive clinical evaluation of candidate biomarkers in multiple trials.

## INTRODUCTION

The outcome of patients with colorectal cancer (CRC) strongly depends on tumor stage at time of diagnosis. Whereas stage I CRC patients have a 5-year overall survival of more than 90%, in stage IV CRC patients it declines to ∼20% or less [1]. When unresectable distant metastases develop, palliative systemic therapy is the only treatment option available to these patients. The backbone of this is a fluoropyrimidine, e.g. capecitabine (CAP) or 5-fluorouracil (5-FU) in combination with either oxaliplatin or irinotecan [2]. Addition of targeted agents directed against vascular epithelial growth factor (VEGF) (bevacizumab) or epidermal growth factor receptor (EGFR) (cetuximab and panitumumab) has been demonstrated to give additional outcome benefit. [3] Only a subset of patients benefit from these regimens, while those patients that do not, may still suffer from considerable toxicity. With the exception of *KRAS/NRAS* mutation status that predicts resistance to EGFR-targeted therapy [4, 5], no other biomarkers exist that adequately predict treatment response in metastatic CRC. Thus, predictive biomarkers are urgently needed to identify the subset of patients who will benefit from a specific treatment.

Hypermethylated genes form a particular category of biomarkers and a number of these have been reported to predict drug response in CRC patients [6, 7]. but inconsistent results for the same markers have been reported [8, 9]. Hypermethylated genes are of particular interest, since DNA methylation is potentially reversible by DNA methyltransferase inhibitors, which could provide a way to restore expression of genes silenced by DNA hypermethylation and thus increase the sensitivity of tumor cells to the agents the gene is associated with [10, 11].

In the present study we set out to identify and validate novel hypermethylated genes that could potentially predict response to treatment with irinotecan in patients with metastatic CRC, using material from two clinical trials, i.e. the Dutch CApecitabine, IRinotecan and Oxaliplatin (CAIRO) study [12] and the Fluorouracil, Oxaliplatin, CPT-11: Use and Sequencing (FOCUS-1) study from the UK [13].

## RESULTS

### Candidate gene selection

Correlation analyses of the DNA methylation status with drug sensitivity in 32 cell lines yielded 22 genes associated with topoisomerase inhibitor-related mode of action. These genes were analyzed for DNA methylation status in the discovery set (n=185). Methylation frequencies ranged from 1% to 98%, average 43% (Table 1).

**Table 1:** Discovery set: observed methylation frequencies of candidate genes

Gene symbol	Methylation frequencyin the discovery set(n=185)
BIK	27%
CAT	14%
CCND2	31%
CDK5	17%
DAPK1	23%
DCR1	39%
EEF1A2 (primer set 1)	1%
EEF1A2 (primer set 2)	5%
HOXA9	40%
IRAK1	40%
LIG4	92%
NUDT1	92%
PAX3 (primer set 1)	85%
PAX3 (primer set 2)	98%
PRKCB1	23%
PROK2	19%
PROP1	93%
PTGS2	8%
RASSF1	14%
RBBP8	45%
RHOB	4%
SPO11	96%
TBX5	96%
TIPARP	19%

### Evaluation of biomarker potential in the discovery set (CAIRO)

**Figure 1:**
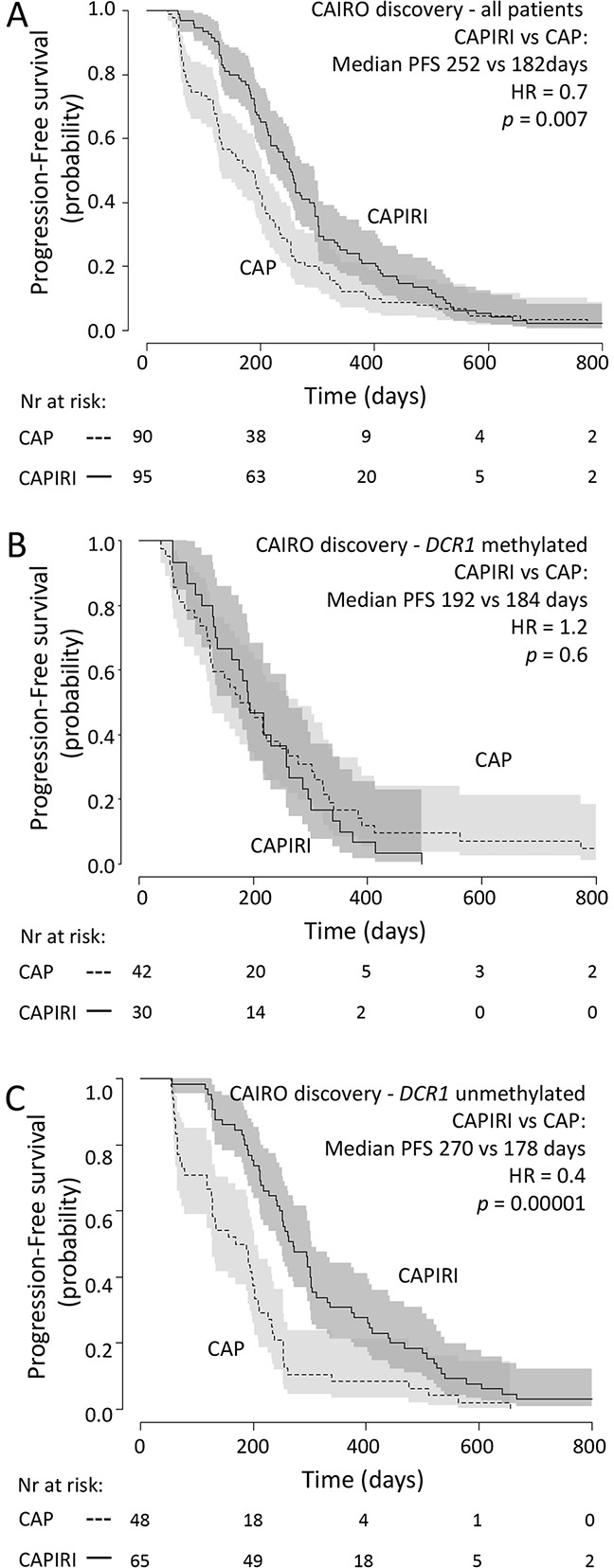
CAIRO discovery set: Progression-free survival Progression free survival in metastatic CRC cancer patients treated in first-line with CAP (dashed line) or CAPIRI (solid line)in (A) all patients from the CAIRO discovery set, in (B) patientswith methylated tumor *DCR1*or in (C) patients with unmethylated tumor *DCR1* 95% confidence interval of the survival probability is shown by dark and light grey shades. HR=Hazard Ratio (CAPIRI versus CAP).

In concordance with the original CAIRO study [12], the sub selection of patients in the discovery set showed significantly longer PFS when treated with CAPIRI (n=95) compared to CAP alone (n=90) (median PFS of 252 vs 182 days for CAPIRI vs CAP, respectively; HR=0.67 (95% CI 0.50-0.90, *p*=0.007) (Figure 1A). *DCR1* was methylated in 72/185 (39%) tumors. To assess the predictive value of each candidate gene, a multivariate survival model was generated including clinical variables, treatment arm, and an interaction term between treatment arm and candidate gene. After correcting for multiple testing, the treatment arm*candidate gene interaction remained significant for Tumor Necrosis Factor Receptor Superfamily member 10c (TNFRSF10c, also known as Decoy Receptor 1 (*DCR1))* and Interleukin-1 Receptor-Associated Kinase 1 (*IRAK1)*. This indicates that the methylation status of these candidate genes exerted an independent effect on PFS that was different between treatment arms (Table 2).

**Table 2:**
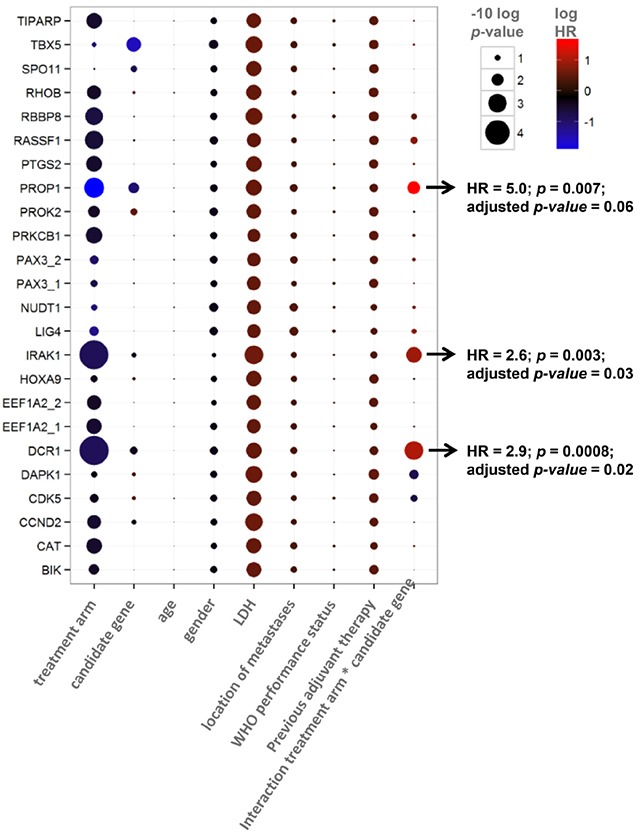
Multivariate analysis for predictive value of candidate genes, showing p-values (size) and Hazard Ratio's (color)

Kaplan-Meyer curve analysis revealed that out of the two final candidate genes, the methylation status of *DCR1* was predictive for PFS after treatment with CAPIRI, but not for PFS after treatment with CAP; patients with methylated *DCR1* tumors progressed more quickly than patients with unmethylated *DCR1* tumors when treated with CAPIRI (HR=2.1 (95% CI 1.3-3.3, *p*=0.001), but no difference was observed between patients with unmethylated or methylated *DCR1* tumors when treated with CAP (HR=0.7 (95% CI 0.5-1.1, *p*=0.1) (Supplementary Figure 1). *IRAK1* methylation was predictive of PFS after treatment with CAP, but not for CAPIRI (Supplementary Figure 1), and hence was not further studied.

Because CAIRO was a randomized controlled trial, we were able to estimate the benefit of CAPIRI treatment over CAP treatment for patients with methylated or unmethylated *DCR1* tumors by comparing PFS between the different treatment arms. Patients with methylated *DCR1* (72 out of 185; 39%) did not benefit from adding irinotecan to CAP (median PFS of 192 vs 184 days for CAPIRI vs CAP, respectively; HR=1.2 (95%CI 0.7-1.9, *p*=0.6; Figure 1B)). In contrast, patients with unmethylated *DCR1* showed a significantly longer PFS when treated with CAPIRI compared to CAP alone (median PFS of 270 vs 178 days for CAPIRI vs CAP, respectively; HR=0.4 (95% CI 0.3-0.6, *p*=0.00001; Figure 1C)).

### Internal validation set (CAIRO)

**Figure 2:**
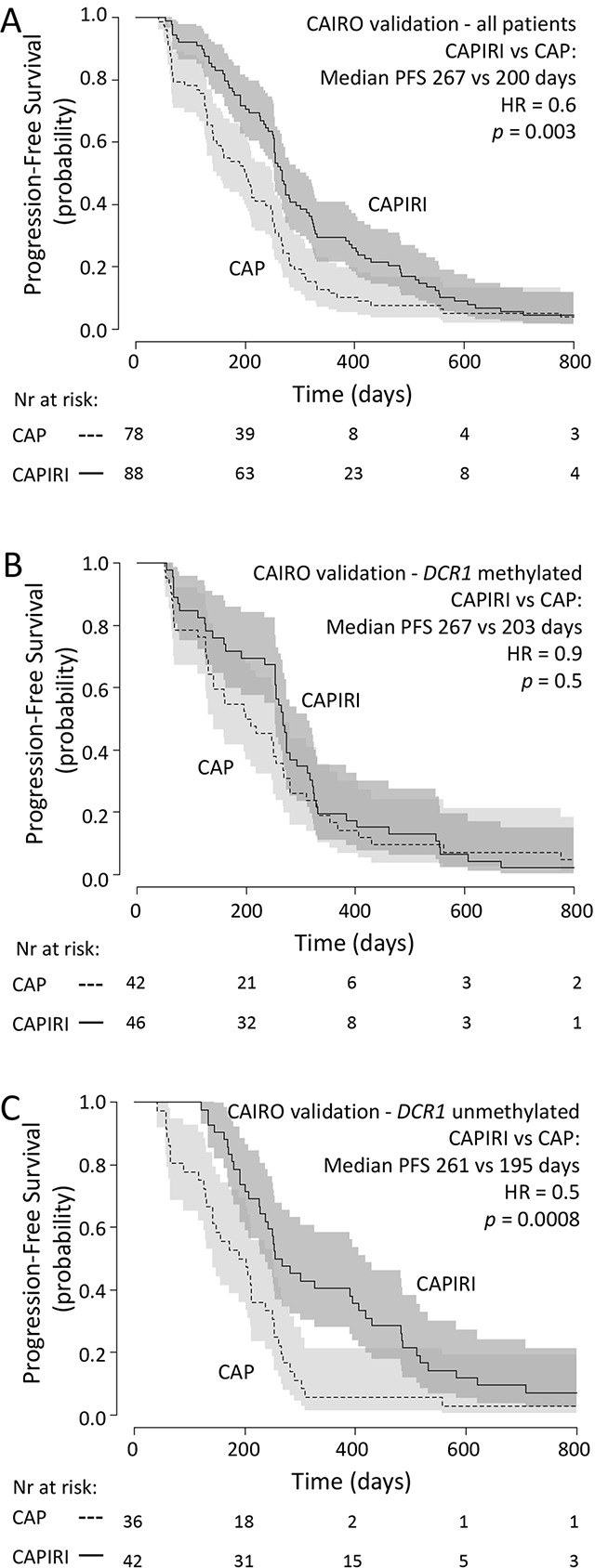
CAIRO validation set: Progression-free survival Progression free survival in metastatic CRC cancer patients treated in first-line with CAP (dashed line) or CAPIRI (solid line)in (A) all patients from the CAIRO validation set, in (B) patientswith methylated tumor *DCR1*or in (C) patients with unmethylated tumor *DCR1* 95% confidence interval of the survival probability is shown by dark and light grey shades. HR=Hazard Ratio (CAPIRI versus CAP).

In the second set of patients from the CAIRO study, in concordance with the original CAIRO study [12], PFS was significantly longer for patients treated with CAPIRI (n=88) compared to patients treated with CAP alone (n=78) (median PFS of 267 vs 200 days for CAPIRI vs CAP, respectively; HR=0.6 (95% CI 0.5-0.9, *p*=0.003; Figure 2A)).

*DCR1* was methylated in 88 out of 166 (53%) tumors. A multivariate analysis, as described for the discovery set, showed a significant interaction between treatment arm and *DCR1* methylation (*p*=0.04, Table 3). Kaplan-Meyer analyses confirmed that patients with methylated *DCR1* tumors did not significantly benefit from CAPIRI treatment over CAP treatment (median PFS of 267 vs 203 days for CAPIRI vs CAP, respectively; HR=0.9 (95%CI 0.6-1.4, *p*=0.5; Figure 2B)), whereas patients with unmethylated *DCR1* tumors did (median PFS of 261 vs 195 days for CAPIRI vs CAP, respectively; HR=0.5 (95%CI 0.3-0.7, *p*=0.0008) (Figure 2C)).

**Table 3:** Evaluation of predictive value of *DCR1* methylation on progression after treatment(multivariate cox proportional hazard model)

Variables in the model	CAIRO discovery set		CAIRO validation set		FOCUS validation set	
	HR	p-value	HR	p-value	HR	p-value
Treatment	0.4	0.00002	0.4	0.0001	0.9	0.4
*DCR1* methylation status	0.6	0.05	0.8	0.3	1.1	0.6
Age	1.0	0.8	1.0	0.03	1.0	0.05
Gender	0.7	0.05	1.0	0.9	0.9	0.5
WHO performance status	1.1	0.5	1.1	0.5	1.0	0.8
Previous adjuvant therapy	1.5	0.04	1.2	0.5	1.1	0.6
LDH	1.7	0.002	1.5	0.02	na	na
location of metastases	1.4	0.07	0.8	0.2	na	na
Interaction treatment*DCR1 methylation status	**2.9**	**0.0008**	**2.0**	**0.04**	**0.8**	**0.3**

HR = hazard ratio

### External validation set (FOCUS)

**Figure 3:**
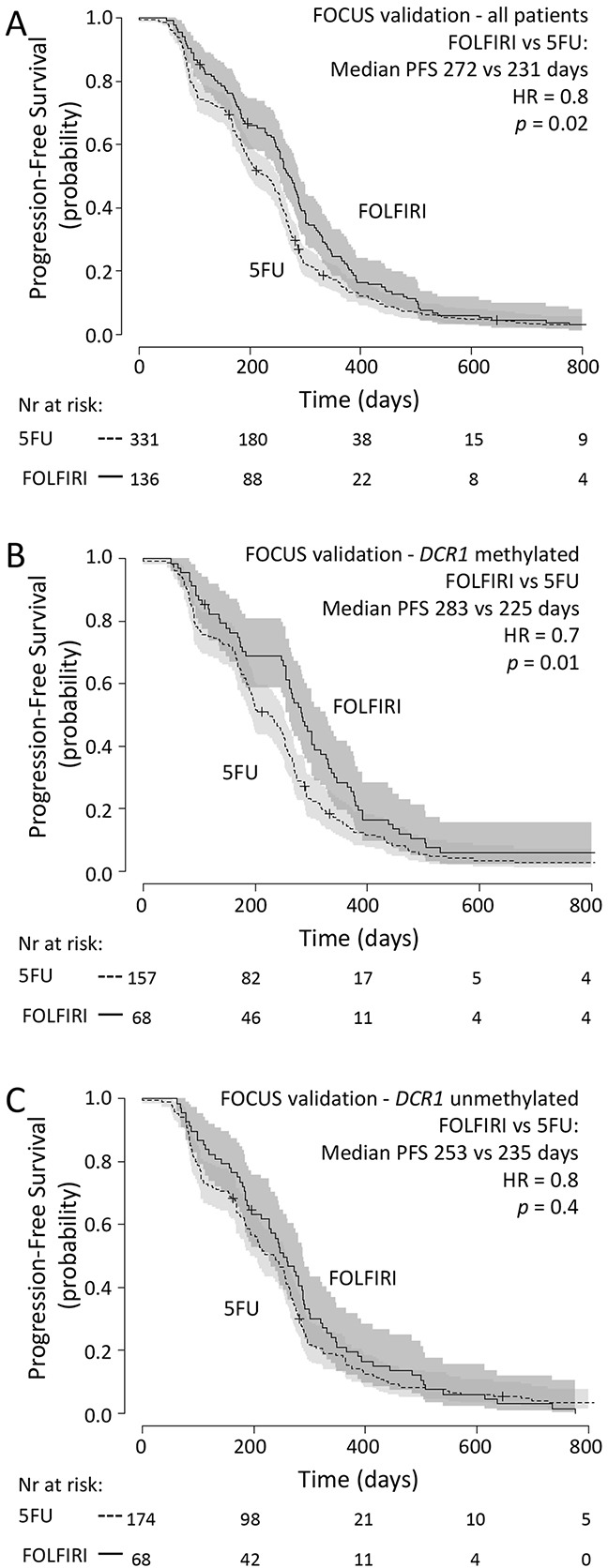
FOCUS validation set: Progression-free survival Progression free survival in metastatic CRC cancer patients treated in first-line with 5-FU (dashed line) or FOLFIRI (solid line)in (A) all patients from the FOCUS validation set, in (B) patientswith methylated tumor *DCR1*or in (C) patients with unmethylated tumor *DCR1* 95% confidence interval of the survival probability is shown by dark and light grey shades. HR=Hazard Ratio (FOLFIRI versus 5-FU).

As an independent validation series, we analyzed 467 tumor samples from another randomized controlled phase III clinical trial (FOCUS) [13]. In this series, similar to the original trial, PFS was significantly longer for patients treated with FOLFIRI (n=136) compared to patients treated with 5-FU alone (n=331) (median PFS of 272 vs 231 days for FOLFIRI vs 5-FU, respectively; HR=0.8 (95%CI 0.6-1.0, *p*=0.02); Figure 3A).

*DCR1* was methylated in 225 out of 467 (48%) tumors. Multivariate analysis revealed that there was no significant interaction between treatment arm and *DCR1* methylation status (*p*=0.3, Table 3). Indeed, Kaplan-Meyer analyses revealed that patients with methylated or unmethylated *DCR1* had a similar effect size from FOLFIRI treatment over 5-FU treatment, as based on HR (methylated *DCR1*: median PFS of 283 vs 225 days for FOLFIRI vs 5-FU, respectively; HR=0.7 (95%CI 0.5-0.9, *p*=0.01) (Figure 3B); unmethylated *DCR1*: median PFS of 253 vs 235 days for FOLFIRI vs 5-FU, respectively; HR=0.8 (95%CI 0.6-1.2, *p*=0.4) (Figure 3C)).

### Methylation of *DCR1* is associated to decreased gene expression

**Figure 4:**
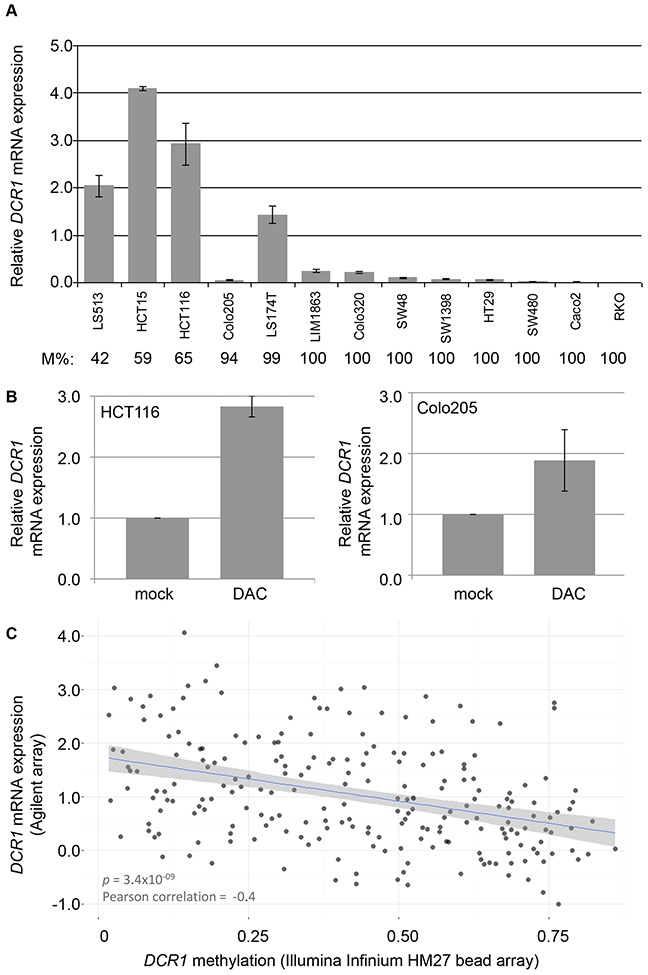
*DCR1* methylation and mRNA expression levels **(A)** *DCR1* mRNA expression analysis in CRC cell lines by RT-PCR. *DCR1* DNA methylation percentage as measured by qMSP (M%) is indicated below each cell line. Quantifications represent mean expression values from three independent experiments. **(B)** *DCR1* mRNA expression analysis by RT-PCR of HCT116 (left panel) and Colo205 (right panel) with and without DAC treatment (*p*=0.005 and *p*=0.08, respectively). **(C)** Scatter plot including a linear regression line and 95% confidence interval, showing the correlation of *DCR1* methylation levels and *DCR1* mRNA expression in 223 CRC tissues from TCGA.

The relation between *DCR1* promoter hypermethylation and gene expression was investigated *in vitro* in a panel of 13 CRC cell lines. Ten out of 13 CRC cell lines were fully methylated for *DCR1* and showed low or absent gene expression. The other three CRC cell lines were hemi-methylated and showed clearly higher gene expression levels (Figure 4A). Treatment of two CRC cell lines, HCT116 and Colo205, with the demethylating agent DAC resulted in increased *DCR1* expression (*p*=0.005 and *p*=0.08, respectively; Figure 4B). In addition, data from The Cancer Genome Atlas (TCGA) database (http://cancergenome.nih.gov), including 223 CRC tumors, confirmed a negative correlation between *DCR1* DNA methylation and *DCR1* mRNA expression (Pearson correlation of −0.4, *p*=3.4E-9; Figure 4C).

## DISCUSSION

In the present study we used a candidate gene approach to identify methylation markers for response to treatment with irinotecan-based therapy. We first made a selection of candidate genes based on *in vitro* findings on their function in relation to the mode of action of irinotecan, i.e. topoisomerase inhibition. Next, we tested for correlation of the methylation status of the candidate genes and PFS after treatment with CAPIRI therapy of metastatic CRC patients participating to the phase III CAIRO trial [12], which identified *DCR1* as a candidate marker. Because patients treated with CAP alone were used as a control group, this analysis showed *DCR1* methylation as a potential negative predictive marker for response to irinotecan-based therapy. The initial finding in the discovery set could be confirmed in a second series of patients from the same CAIRO study, which indicated that the initial finding was not a stochastic statistical finding. However, validation in a second, independent series of metastatic CRC patients from the phase III FOCUS trial [13], treated with first-line FOLFIRI or 5-FU alone, did not confirm *DCR1* methylation status as negative predictive marker for response to irinotecan-based therapy.

Developing predictive biomarkers that reach the phase of introduction into clinical practice has proven to be highly challenging. The literature is full of proof of concept publications on potential biomarkers, but in most instances no further validation follows or if so they fail to publish. The current study was carefully designed in order to overcome most common pitfalls in biomarker discovery [14, 15]; i.e. a strong biological rationale existed for the preselected candidate genes, and extensive evaluation (discovery, internal validation and external validation) was performed in a prospective-retrospective design [16] on a total of 818 archival tumor samples derived from two similar well-conducted phase III randomized clinical trials, providing the highest quality of clinical annotation [12, 13] In addition, both clinical trials included a control group (i.e. CAP as control group for CAPIRI and 5-FU as control group for FOLFIRI), which is required to distinguish predictive from prognostic markers. Furthermore, biomarker independence was tested by including potential confounding factors in the statistical models. Nonetheless, after initial validation in a second sub-sample of the CAIRO study, we could not validate *DCR1* methylation as a negative predictive marker for response to irinotecan-based therapy in the independent patient series from FOCUS. A lack of correlation between *DCR1* methylation and *DCR1* gene expression could be one of the reasons why *DCR1* methylation as a marker for response to irinotecan-based therapy did not validate. However, our cell-line experiments as well as analysis of a large series from the TCGA database did show a correlation between *DCR1* DNA methylation and gene expression silencing. All this data together would suggest to simply discard *DCR1* methylation as a potential biomarker for response to irinotecan-based therapy, although our findings could still be otherwise explained. The two trials for instance, while they show substantial resemblances at first glance, differ in a number of features related to inclusion (e.g. the performance scores leading to differences in patient characteristics), population differences (methylation varies between populations and races [17]) and importantly treatment (e.g. different backbone treatment; CAP versus 5-FU). 5-FU, in contrast to CAP, is given in combination with Leucovorin, which is a reduced folic acid. Folic acid is implicated in regulation of DNA methylation and aberrant folic acid levels may affect global or site-specific DNA methylation levels in tissues [18, 19]. Treatment with Leucovorin thus could potentially affect DNA methylation levels in tumor tissue over time and thereby influencing the association with an a priori measured DNA methylation marker and drug sensitivity.

The current study has some limitations. For example, the drug-response screen on candidate genes was performed on cell lines rather than on actual human tumor samples. Although the use of cell lines reduces the number of confounding factors as compared to human tumor samples, it remains a challenge to translate the relevance of the observed data to the *in vivo* situation [20], and other interesting genes might have been missed by this approach.

Another limitation of the current study is that measurements were performed on samples from the primary tumor, while patients were treated for their metastases, raising the question whether intra tumor heterogeneity could play a role. Although metastases can acquire additional genomic alterations, they keep most of the alterations present in the primary tumor [21, 22]. As DNA methylation is usually an early event in colorectal carcinogenesis, this is likely to be the case here as well [23].

Lastly, *DCR1* methylation analyses were performed with identical primers but with different reagents in different laboratories for the three study cohorts. This could have introduced variability in test results. The proportion of patients having a positive test result was slightly different for the three cohorts indeed (39% in the discovery set, 53% in the internal validation set and 48% in the external validation set). However, because the predictive value of *DCR1* methylation with regard to irinotecan-based therapy showed similar results in the two cohorts with largest relative difference in prevalence of methylation (39% vs 53%), this variability is not likely to be the cause of the inability to validate *DCR1* methylation as a predictive biomarker.

Another challenge is the biological complexity underlying tumor response to treatments and the question if a single biomarker can capture responses of a system as a whole. Moreover, the biology of DCR1, which acts in the TRAIL pathway, is complex. In normal tissue DCR1 is thought to work as a decoy receptor for the TNF-related apoptosis-inducing ligand (TRAIL), thus having an anti-apoptotic effect in the TRAIL pathway [24]. There are some indications, however, that TRAIL receptors in cancer have an anti-apoptotic role via the NF-кB survival pathway and hence silencing of *DCR1* would subject a cancer cell to a pro-survival and pro-growth signal [25, 26]. The latter supports our finding in the CAIRO study that patients with a methylated *DCR1* tumor have a worse outcome. Although our candidate gene selection revealed that *DCR1* methylation is associated with sensitivity to topoisomerase inhibitor-related mode of action, the specific functional role of *DCR1* methylation with regard to irinotecan is not known. It would be interesting to study this in colorectal cancer but because we could not validate *DCR1* methylation as predictive marker for irinotecan treatment response, we feel that functional experimental analyses to dissect the role of *DCR1* methylation and its interaction with irinotecan treatment are beyond the scope of the present study.

In conclusion, *DCR1* methylation status was identified as a predictive marker for irinotecan-based therapy in metastatic colorectal cancer in both a discovery and an initial validation set. This could not be confirmed in an external validation data set, for which the difference in backbone treatments (5FU vs capecitabine) may possibly be an explanation. The present study highlights the challenge and importance of extensive evaluation of potential biomarkers. It also shows the complexity and extensiveness of systematic evaluation of a potential biomarker in order to generate more than just a proof of concept, and that a well-designed study is not a guarantee of success. Improvements in multi-team collaborations and in organizing data acquisition and biobanking in clinical trials will be necessary for efficient and successful discovery of predictive biomarkers in the future.

## MATERIALS AND METHODS

### Candidate gene selection

Candidate gene selection was based on correlations between methylation of 389 genes involved in DNA Damage Repair and Response and drug response in 32 cell lines, which is described in detail in the supplementary information. Because irinotecan is a topoisomerase-I inhibitor, genes associated sensitivity to topoisomerase inhibitor-related mode of action were considered.

### Patient sample selection

Patients selected for the current study participated in either of two phase III trials, namely the CApecitabine, IRinotecan and Oxaliplatin (CAIRO) study of the Dutch Colorectal Cancer Group (DCCG) (CKTO 2002-07, ClinicalTrials.gov; NCT00312000) [12], and the Medical Research Council Fluorouracil, Oxaliplatin, CPT-11: Use and Sequencing (FOCUS) study (ISRCTN 79877428) under the auspices of the United Kingdom National Cancer Research Institute Colorectal Cancer Studies Group [13]. Written informed consent was required from all patients before study entry, and included consent for translational research on tumor tissue.

### CAIRO biomarker populations

In the CAIRO study, 820 patients were randomized between sequential (arm-A, first-line CAP, second-line irinotecan and third-line CAPOX) and combination treatment (arm-B, first-line CAPIRI and second-line CAPOX). Patient and sample selection for molecular research purposes have been described before [27]. In short, we selected patients of whom FFPE tissue of the primary tumor was available through the Dutch national pathology registry PALGA [28]. Inherently these are the patients who underwent resection of the primary tumor (n=633), of which tissue samples were available for 478 patients. To be sure that the effect that we observed on outcome was related to the response to treatment and not to an unrelated intrinsic prognostic factor, patients that received only 1 or 2 cycles of therapy were excluded. In order to prevent that we would lose early progressors that could still be related to treatment, we did include those patients that received 2 cycles when death followed due to progressive disease. These selection criteria left us with 425 tissue samples for further analysis. Finally, only tumors containing an area of at least 70% tumor cells were selected for DNA extraction, leaving 351 tissue samples for further analysis DNA. The CAIRO discovery was done on a series of samples available that had been used in a previous study [27]. This series consisted of 185 patients, of which 90 patients were treated with first-line capecitabine (CAP) and 95 were treated with first-line capecitabine plus irinotecan (CAPIRI). The patient samples were matched according to the stratification factors in the original study (for the subgroup of patients that underwent resection of the primary tumor, since these are the patients from whom material was available to be included in this study) [12], that is, performance status, predominant metastatic site, previous adjuvant therapy and serum lactate dehydrogenase level (LDH).

For the initial validation set, patients were selected, with no further criteria, from the remaining patients of which tumor DNA samples were available. These comprised 166 patients, of which 78 were treated with first-line CAP and 88 were treated with first-line CAPIRI.

### FOCUS biomarker validation population

In the FOCUS study, 2135 patients without pretreatment were randomly assigned to three treatment strategies in the ratio 1:1:1. In strategy A (control group) patients received first-line 5FU, followed by second-line irinotecan. In strategy B patients received first-line 5FU, followed by second-line 5-FU plus irinotecan (FOLFIRI; Strategy B-ir) or 5-FU plus oxaliplatin (FOLFOX; strategy B-ox). In strategy C patients received FOLFIRI (C-ir) or FOLFOX (C-ox) from the outset. For the current study, patients from strategy A (first-line 5FU), strategy B-ir (first-line 5FU) and strategy C-ir (first-line FOLFIRI) were selected, for which a total of 515 tumor DNA samples were available. From these, patients treated with at least three cycles of first-line therapy were selected, leaving 467 samples. These came from 331 patients treated with at least three cycles of first-line 5-FU (249 from strategy A and 82 from strategy B-ir) and 136 patients treated with at least three cycles of first-line 5-FU plus irinotecan (FOLFIRI; strategy C-ir).

### DNA isolation and methylation analysis

Tumor samples from the CAIRO trial were retrieved through the Dutch national pathology registry PALGA [28] DNA was extracted from formalin-fixed paraffin-embedded tissue samples of primary tumors, resected before chemotherapy, as described before. [29, 30] DNA concentrations were quantified using the Nanodrop 1000 UV spectrophotometer (Nanodrop Technologies Inc, Wilmington, DE, USA). DNA was subjected to sodium bisulfite conversion using the EZ DNA Methylation Kit (Zymo Research, Orange, CA, USA) according to the manufacturer's protocol.

Tumor DNA from the FOCUS trial was extracted as described in 7 [29]. DNA samples were subsequently cleaned by ethanol precipitation and DNA concentrations were quantified using the Nanodrop 1000 UV spectrophotometer (Nanodrop Technologies Inc).

All methylation assays were performed blind to information on treatment or survival outcome. The CAIRO discovery set was subjected to high-throughput LightCycler MSP assay (LightCycler 480 SYBR Green I Master kit (Roche, Vilvoorde, Belgium)) for the 22 selected candidate genes. Primers were designed to promoter regions (i.e. −1000 to +200 bp relative to the transcription start site). Primers from the literature were used when they experimentally passed our quality control; see Table 4 for primer sequences. Quality control was performed with *in vitro* Methylated DNA (Chemicon, Temecula, CA) as a positive control and DNA from the unmethylated human HCT116 DKO cell line as a negative control. Per sample, 20 ng bisulfite-modified DNA was amplified with the following PCR conditions: 95°C for 10 minutes followed by 45 cycles of 95°C for 10 seconds, 60°C for 30 seconds and 72°C for one second. Amplification of beta-actin was used as an unmethylated reference gene. The amplicons were checked for size and quantified by capillary electrophoresis (LC90 Labchip; Caliper Lifesciences).

**Table 4:** MSP primer sequences

Name	Lightcycler MSP primers	
	S primer	AS primer
BIK	TTTTTGGAGTTTCGGTTTTTAC	CTTTACACGAATAACCTCCGTTC
CAT	GTTTGTTGTTTCGAGTTCGTG	ATCTTAACCTACCTAACGCCGA
CCND2	CGGGGTTGTTTTATTCGTATCG	CAACCAACTTACGTCACCGCT
CDK5	AGTTTTGCGGGAAATGTTAATAC	AAACTCCGATCTCAACAACGA
DAPK1	TAAGGAGTCGAGAGGTTGTTTC	CCTACCGCTACGAATTACCGA
DCR1	TTACGCGTACGAATTTAGTTAAC	CATCAAACGACCGAAACG
EEF1A2 (1)	GTTCGTGATTAGTAGAGTCGGGT	ACAACGAATAAAAATAAAACGCC
EEF1A2 (2)	TTAGGTTGGGTACGTTCGTGA	ACAACGAATAAAAATAAAACGCC
HOXA9	AGGAGCGTATGTATTTGTCGTTC	AACGCTATACCCGCTACGATA
IRAK1	AGGATGTGTACGAGGTCGGTT	CGAACTACGACTATACGAACGCT
LIG4	GAGTTAAAAACGGGAGAAATCGT	CACAACGCTATAAACTACGCC
NUDT1	GTATTTTTCGAGTTCGTTACGTTT	TCCTCTTAACGTCCAACGAC
PAX3 (1)	TTTGGGTATAGCGTCGGTT	ATTCCCGAAAATCATCCGC
PAX3 (2)	ATAGTTTTCGAGGGTTATTCGC	CCTAAACACAACGCCGACC
PRKCB1	GTATCGCGTTTAGGTTTCGTTT	CCGACGCTACAAAACTACGA
PROK2	ATAAAGGTTAGTTTCGTCGTGA	ACACGTACTCGTCTAAAAACCG
PROP1	CGAGTTATGGAAGTAGAAAGGAGGC	ATAATCGAAATCCCAATAACCGA
PTGS2	TTACGGAAATGAGAAAATCGG	GCCTAAAACGATAAAACTCGAAA
RASSF1	GCGTATTGTAGGTTTTTGCGT	TAATCCCTAACCGTAACCACCG
RBBP8	GTATTTTTATACGGGTAAGGCGA	TACCCCGCTACTCTACTCCGC
RHOB	AGGAGGGGATTCGGGTATC	TAATTAACGACCCAAACCG
SPO11	AGTGTGGGTCGCGTAGGTATC	CTAAATCCAATATCCGCAACACG
TBX5	TCGGTATTGATAGGCGAAGAC	CTATAAAACTTAAAAACGTCACGAA
TIPARP	TAAGGTTTACGAAATAGTCGGTC	ACTACCACCAAACGAAATCGC
Beta Actin	TAGGGAGTATATAGGTTGGGGAAGTT	AACACACAATAACAAACACAAATTCAC
**Name**	**qMSP primers**	
	**S primer**	**AS primer**
Beta Actin	TGGTGATGGAGGAGGTTTAGTAAGT	GAATTTTTTTATGTGTATGAATTTAGTTAAT
DCR1_U	GAATTTTTTTATGTGTATGAATTTAGTTAAT	CCATCAAACAACCAAAACA
DCR1_M	TTACGCGTACGAATTTAGTTAAC	CATCAAACGACCGAAACG

For the CAIRO validation set and CRC cell lines a quantitative MSP (qMSP) assay for *DCR1* was used. The primers for methylated DNA were the same primer sequences as the primers used for LightCycler analyses described above and were designed at the exact location as described before [31]. qMSP reactions were carried out in duplicate in 25 μl reaction volumes, each containing 36 ng of bisulfite-treated DNA, 10 pmol of each primer and 1x Power SYBR Green PCR Master Mix (Applied Biosystems, Foster City, CA). Each plate included no template controls and a standard curve with a serial dilution of bisulfite-modified DNA from a mixture of methylated cell line (HCT116) and unmethylated cell line (HCT116-DKO). PCR conditions were 95°C for 15 minutes, followed by 40 cycles at 95°C for 30 seconds, 56°C for 30 seconds and 72°C for 30 seconds, followed by a melt curve stage to check the specificity of the amplification reaction. Cycle threshold (Ct) values were measured at a fixed fluorescence threshold, which was always in the exponential phase of the amplification curves. The methylation percentage per sample was calculated according to the formula 2e-[mean CtM-reaction]/(2e–[mean CtM-reaction]+2e-[mean CtU-reaction])x100. The U (unmethylated) and M (methylated) reactions were amplified with comparable efficiencies. A sample was called methylated when the methylation percentage was higher than observed in a panel of 21 normal colon mucosa from non-cancer patients as measured in triplicate (median plus two times the standard deviation = 6%).

The FOCUS validation set was analyzed with a qMSP assay for DCR1 as well. The primers for methylated DNA were equal to the primers used in the CAIRO discovery and validation study. qMSP reactions were carried out using a 7500 Fast Real-Time PCR System (Applied Biosystem,) in duplicate in 25 μl reaction volumes, where each reaction contained 40 ng of bisulfite-treated DNA, 10 pmol of each primer and 12·5 μl SYBR Green PCR Master Mix (Applied Biosystems). Each plate included no-template controls and a standard curve with a serial dilution of bisulfite-modified DNA prepared from *in vitro* Methylated DNA (Chemicon). PCR conditions were 95°C for 15 minutes, 40 cycles at 95°C for 15 seconds, 60°C for 60 seconds, followed by melt curve analysis to check the specificity of the amplification reaction. Amplification of beta-actin was used as an unmethylated reference gene, using the same PCR conditions. The Ct ratio per sample was calculated according to the formula 2e-[mean CtDCR1 – mean CtACTB]. A sample was called methylated when the Ct ratio was higher than observed in a panel of 22 normal colon mucosa from non-cancer patients as measured in duplicate (median plus two times the standard deviation = 0·006)

### Cell lines

HCT15, HCT116, LS513, LS174T, Colo320, SW48, SW1398, HT29, Colo205, SW480, and RKO were cultured in Dulbecco's modified Eagle's medium (DMEM; Lonza Biowhittaker, Verviers, Belgium) containing 10% fetal bovine serum (Hyclone, Perbio, UK). Caco-2 was cultured in RPMI 1640 (Lonza Biowhittaker) containing 20% fetal bovine serum. LIM1863 was cultured in RPMI 1640 (Lonza Biowhittaker) containing 5% FCS, 0·01 mg/ml thioglycerol, 1 mg/ml insulin and 1 μg/ml hydrocortisone. All cell culture media were supplemented with 2 mM L-glutamine, 100 IU/ml sodium penicillin (Astellas Pharma B.V., Leiderdorp, The Netherlands) and 100 mg/ml streptomycin (Fisiopharma, Palomonta (SA), Italy). To investigate re-expression of *DCR1* after inhibition of DNA metyltransferases, HCT116 and Colo205 cells were treated with 5000 nM 5-aza-2′-deoxycytidine for three days (DAC, Sigma Chemical Co., St. Louis, MO, USA).

### RNA isolation and qRT-PCR

Total RNA was isolated from cell lines using TriZol reagent (Invitrogen, Breda, the Netherlands), and subjected to purification using RNeasy Mini Kit (Qiagen). After DNAse treatment (RQ1 DNAse, Promega, Leiden, the Netherlands), cDNA was made with the Iscript cDNA Synthesis Kit (BioRad, Veenendaal, the Netherlands). Quantitative RT-PCR was done using TaqMan® Gene Expression Assays from Applied Biosystems directed to *DCR1* (Hs00182570_m1) and a reference gene *B2M* (Hs00984230_m1). Relative expression levels were determined by calculating the Ct-ratio (Ct ratio = 2e-(Ct*^DCR1^*–Ct*^B2M^*))x1000.

### TCGA data

*DCR1* DNA methylation (Illumina Infinium HM27 bead array; HM27) and mRNA expression (Agilent array) data were obtained via cBioPortal for Cancer Genomics (http://www.cbioportal.org; [32]) on 223 CRC tumors included in The Cancer Genome Atlas (TCGA) Colorectal Cancer project. This data set was downloaded on the 14th of July 2015 from all tumors with available methylation and mRNA expression data from the Colorectal Adenocarcinoma (TCGA, Nature 2012) dataset [33].

### Statistical analysis

The primary endpoint of the present study was progression free survival (PFS) under first-line systemic therapy with or without irinotecan. PFS for first-line treatment was calculated from the date of randomization to the first observation of disease progression or death reported after first-line treatment. To test the predictive value of candidate genes, multivariate Cox proportional hazard models were built that included the variables treatment, candidate gene and an interaction term treatment*candidate gene. In the CAIRO as well as the FOCUS trial, patients were randomized between treatment arms, which resulted in similar clinical characteristics between the treatment arms. However, because we were analyzing patient subsets from the original trials, for *DCR1*-specific analyses we corrected the estimates of predictive value for those variables that could have possible prognostic effect, by including them in the multivariate analyses. These were age, gender, WHO performance status and prior adjuvant therapy for both the CAIRO and the FOCUS samples, plus normal or abnormal LDH and location of metastases for CAIRO. Cox proportional hazard models were used to estimate Hazard Ratios (HR) and 95% confidence intervals (CI). Kaplan-Meier analyses and log-rank tests were used to estimate survival over time. Correction for multiple testing in the discovery set was done by the Benjamini Hochberg method.

Student's T-test was used for comparison of *DCR1* expression levels before and after DAC treatment of HCT116 and Colo205. Pearson correlation analysis was used to measure correlation between *DCR1* methylation and mRNA expression levels from 223 primary CRC tissue samples as provided by The Cancer Genome Atlas (TCGA) database.

Statistical analyses were performed using the computing environment R version 3.2 [34], including the packages *survival* and *rms* [35–37].

## SUPPLEMENTARY FIGURE



## Abbreviations

CRC: Colorectal Cancer
TNFRSF10C: Tumor Necrosis Factor Receptor Super Family 10C
DCR1: Decoy Receptor 1
MSP: Methylation Specific PCR
PFS: Progression Free Survival
CAIRO trial: Capecitabine Irinotecan and Oxaliplatin trial
FOCUS trial: Fluorouracil, Oxaliplatin, CPT-11: Use and Sequencing study
MOA: common modes of action
QC: quality control
Ct: Cycle threshold
DAC: 5-aza-2′-deoxycytidine
