# Supplementary material for: Decoy receptor 1 (DCR1) promoter hypermethylation and response to irinotecan in metastatic colorectal cancer
Source: Oncotarget. 2017 Jun 27;8(38):63140–54. doi: 10.18632/oncotarget.18702 (PMC5609910; doi:10.18632/oncotarget.18702)
Supplement: Supplementary file 1 [file oncotarget-08-63140-s001.pdf]

Decoy receptor 1 (DCR1) promoter hypermethylation and response to irinotecan in metastatic colorectal cancer

SUPPLEMENTARY MATERIALS

Analysis, selection procedure of candidate genes: correlation between methylation status and predictivity and sensitivity to drug response

Drug activity data sets are publicly available from a number of sources. Here, methylation data for a number of DNA markers was correlated to drug activity data provided by The Genomics and Bioinformatics Group, 2000 Publications Data Set, Drug Activity of 118 —Mechanism of Action Drugs (MOA's), available at its website http://discover.nci.nih.gov/nature2000/data/selected_data/dataviewer.jsp?baseFileName=a_matrix118.

To generate the methylation data, 1156 assays were tested against 32 cell lines from breast cancer (BT549, HS578T, MCF7, MDAMB231, T47D), colon cancer (Colo205, HCT116, HCT15, HT29, SW620), lung cancer (A549, H226, H23, H460, H522), leukemia (CCRF-CEM, HL60, K563, MOLT4, RPMI8226, SR), melanoma (MALME3M, SK-MEL2, SK-MEL5, SK-MEL28), ovarian cancer (OVCAR3, SKOV3), prostate cancer (DU145, PC3) and renal cancer (7860, A498). The 1156 assays were designed to cover the TSS proximal CpG island of 631 genes involved in DDR (DNA Damage Repair and Response). Of the 1156 assays tested, 562 assays (389 genes) were retained for which we observed at least one methylated and one unmethylated cell line sample. For the same set of 32 cell lines the -log(GI50) scores of 118 drugs from the NCI60 database were selected. These drugs were grouped into 15 MOA's.

The above datasets were combined to correlate the methylation profile of 562 assays to the activity profile of 118 drugs and 15 MOA's. For each of the 562x118 couples (assay,drug) and the 562x15 couples (assay,MOA) a p-value was computed via randomization.

Given a couple (assay,drug) or (assay,MOA), the methylation profile of the assay was used as a starting point for the randomization experiment. This profile divides the set of cell lines in methylated and unmethylated ones (cell lines where the methylation call is missing were ignored). For both subsets of cell lines the average -log(GI50) score of the drug (or MOA): avgM(-log(GI50)) and avgU(-log(GI50)) was computed. The larger the difference between both averages, the more predictive the assay is of sensitivity to the drug (or MOA). If avgM > avgU it was assumed that methylation indicated higher sensitivity and the difference as avgM-avgU was computed. Otherwise we assumed the unmethylated state indicated higher sensitivity and computed the difference as avgU-avgM.

Using difference avgM-avgU or avgU-avgM as a reference, a randomization experiment consisting of ten million iterations was conducted. In each iteration a stratified sample from the 32 cell lines was selected, the difference between the average -log(GI50) in selected and unselected cell lines was computed, and it was counted how often this difference was at least as high as the reference difference, and the result was divided by ten million to obtain a p-value. The stratified sampling strategy was based on the categorization of the 32 cell lines into eight subtypes: breast (n=5), colon (n=5), leukemia (n=6), lung (n=5), melanoma (n=4), ovarian (n=2), prostate (n=2) and renal (n=3). To compose a random sample, we randomly selected within each subtype the number of methylated (in case avgM>avgU) or unmethylated (in case avgU>avgM) cell lines within that subtype. This was done to favor markers that discriminate between high and low sensitivity within different tissue types.

Robust assays were identified and selected. Those assays are highly predictive for the response of cell lines to single drug or to a group of drugs with a common mode of action. The mode of action taken into consideration for the present study was topoisomerase inhibitors.

Quality control was performed using in vitro methylated DNA sample, unmethylated DNA sample and no template control sample (H2O). From the Lightcycler platform, the cycle threshold (ct) and melting temperature (Tm) Calling are calculated by the Roche Lightcycler 480 software (Software release 1.5.0). From the capillary electrophoresis platform, the band sizes and band heights are calculated by the Caliper software (Caliper Labchip HT version 2.5.0 , Build 195 Service Pack 2).

In a first stage, the melting temperature and product size of in vitro methylated DNA are measured for a marker. A sample is called positive for that marker if the melting temperature and product size are within the specified boundaries of a measured in vitro methylated reference. Additional rules are imposed on the Ct value and the band intensity of the product with the right size. Product size has to be within the reference product size +/- 10 bp interval. Melting temperature has to be within the reference product temperature +/- 2 degrees Celsius range. In addition, the cycle threshold has to be under 40 cycles and the correct band intensity height has to be higher than 20, the latter is a relative number calculated by the caliper software.

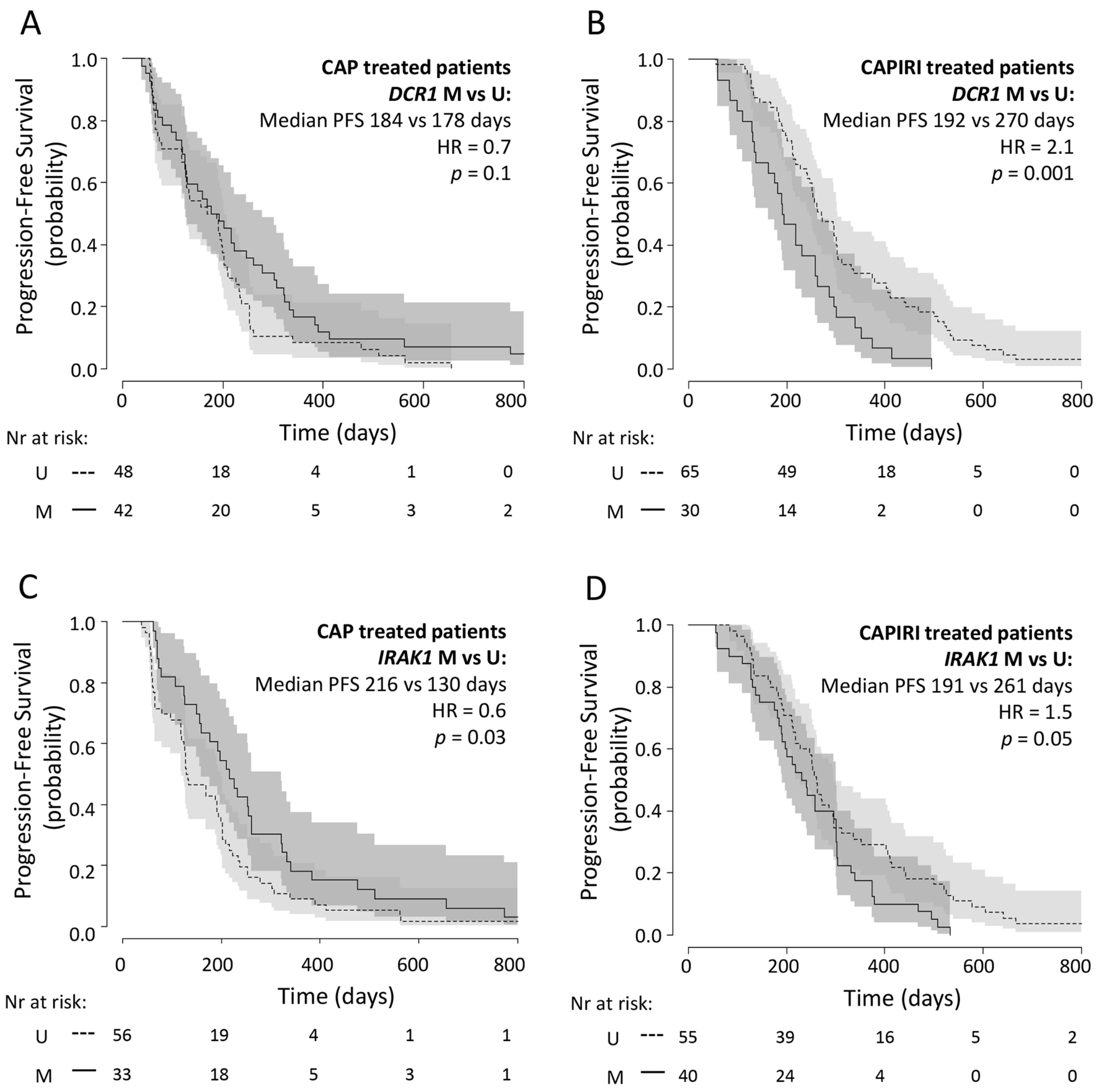

Supplementary Figure 1: CAIRO discovery set: Progression-free survival stratified for *DCR1* or *IRAK1* methylation status. Progression free survival in metastatic colorectal cancer patients with unmethylated (U; dashed lines) or methylated (M; solid lines) DCR1 **(A and B)** or IRAK1 **(B and C**) treated in first line with CAP (**A and C**) or CAPIRI (**B and D**). HR=Hazard Ratio (M versus U). 95% confidence interval of the survival probability is shown by dark and light grey shades.HR=Hazard Ratio (M versus U).
